# Adopting Stimulus Detection Tasks for Cognitive Workload Assessment: Some Considerations

**DOI:** 10.1177/00187208241228049

**Published:** 2024-01-21

**Authors:** Francesco N. Biondi

**Affiliations:** 1 8637 University of Windsor, Canada; 2 172839 University of Utah, USA

**Keywords:** stimulus detection task, psychomotor vigilance task, detection response task, ISO DRT, PVT, workload, vigilance, response times, automation, automated driving systems, Fatigue, Drowsiness

## Abstract

**Objective:**

This article tackles the issue of correct data interpretation when using stimulus detection tasks for determining the operator’s workload.

**Background:**

Stimulus detection tasks are a relative simple and inexpensive means of measuring the operator’s state. While stimulus detection tasks may be better geared to measure conditions of high workload, adopting this approach for the assessment of low workload may be more problematic.

**Method:**

This mini-review details the use of common stimulus detection tasks and their contributions to the Human Factors practice. It also borrows from the conceptual framework of the inverted-U shape model to discuss the issue of data interpretation.

**Results:**

The evidence being discussed here highlights a clear limitation of stimulus detection task paradigms.

**Conclusion:**

There is an inherent risk in using a unidimensional tool like stimulus detection tasks as the primary source of information for determining the operator’s psychophysiological state.

**Application:**

Two recommendations are put forward to Human Factors researchers and practitioners dealing with the interpretation conundrum of dealing with stimulus detection tasks.

## The Problem at Hand

Stimulus detection tasks offer a simple yet effective means of measuring the user’s workload during task completion. Considering the unidimensional nature of this paradigm whereby nonoptimal workload—no matter its nature—results in a task performance decline, understanding the etiology of the observed data pattern is key to a correct data interpretation. Whereas this may be easier in conditions like multitasking wherein the decline in task performance is likely the direct result of the added task load (Strayer & Cooper, 2015), there are key applications where data interpretation is murkier. For example, take those situations wherein the human operator hands over some (but not all) operations to an automated system while concurrently maintaining responsibility for the task at hand. While it is arguable that the partial transfer of control will result in a *reduction* in workload and, thus, boredom (Parasuraman & Manzey, 2010), it can be posited that the additional cognitive cost of maintaining vigilance over the automated system’s functioning will instead *increase* workload (Warm et al., 2008). No matter the real cause, both conditions will result in a decline in detection task performance.

This mini-review investigates the germane issue of data interpretation when using stimulus detection tasks as a way to measure workload during task completion in the workplace. The analysis begins with a discussion of the importance of measuring the user’s state in the Human Factors practice, and the contribution made by stimulus detection tasks. The issue of the interpretation conundrum is then analyzed in the context of the inverted-U conceptual model. Finally, practical recommendations for practitioners and researchers are put forward.

## The Importance of Workload Assessment

Maintaining an optimal psychophysiological state is necessary for task performance and safety. Research across the Human Factors and Ergonomics spectrum highlight how workload declines during the execution of a task lead to decrements in response times and accuracy and increase the risk of accidents and injuries. For example, failures in sustaining attention toward mission-relevant tasks is often associated with slower responses and a greater chance of overlooking safety-critical information (Biondi et al., 2015; Cummings et al., 2013; MackWorth, 1948; Parasuraman et al., 2009). Conditions of underload like fatigue are known contributors to workplace injuries among law enforcement officers (Fekedulegn et al., 2017) and healthcare professionals (Patterson et al., 2012). Likewise, conditions of overload resulting from, for example, multitasking and distraction, are also contributing factors to workplace errors and accidents (Bonsang & Caroli, 2021; Lee, 2008).

Key to maintaining optimal workload is employing effective means of measuring it. Practitioners have adopted a variety of methodologies for monitoring the user’s state during task completion. Physiological metrics like heart rate and skin conductance are often used as metrics of fatigue (O’Keeffe et al., 2020; Segerstrom & Nes, 2007). Methodologies such as event-related potentials (ERPs) and functional near-infrared spectroscopy (fNIRS) have also been used to determine levels of attentional processing during controlled experiments (Aghajani et al., 2017; Perello-march et al., 2021; Zhu et al., 2021). These approaches have clear advantages in their ability to provide an accurate real-time tracking of the operator’s state, often with a high degree of temporal resolution. However, their adoption likely require the use of scientific-grade sensors and laborious data processing which may limit their employment in the real-world scenarios.

## Stimulus Detection Tasks in Human Factors

Stimulus detection tasks represent a cheaper, simpler means for detecting the operator’s state in the workplace. Their simple design requires responding to the presentation of an intermittent stimulus, auditory, visual, or vibrotactile in nature, via pressing an actuator like a key on a keyboard or a microswitch. The amount of time taken to respond to the stimulus and the number of accurate detections provide information on the operator’s state (e.g., vigilance and workload) so that faster responses and higher accuracy are indicative of their greater ability to maintain optimal performance in the primary task at hand.

Two common examples of stimulus detection tasks in Human Factors are the psychomotor vigilance task (PVT) and the detection response task (DRT). The PVT was conceived to measure vigilance in tasks requiring sustained attention (Graeber et al., 1990; Kribbs et al., 1993). This task requires responding to the intermittent onset of a stimulus which is presented with a variable interstimulus interval of 2–10 seconds. It is also administered alone, not in conjunction with any other task, to measure vigilance decrements induced by, for example, prolonged drowsiness. It typically has a set duration of 10 minutes, which may make it less suitable at tracking temporal fluctuations in vigilance during prolonged supervisory tasks. Hogan (1966) adopted it to measure interindividual differences in vigilance performance. Graeber et al. (1990) employed it to quantify drowsiness in aircraft pilots. In the years since, it has been widely adopted to quantify losses in vigilance resulting from drowsiness. Thomann et al. (2014) presented participants with a visual stimulus on a computer screen to which they were asked to respond by pressing a button. When compared to control sample, participants suffering from daytime sleepiness displayed longer response times and lower accuracy (see Drummond et al., 2005 for consistent patterns). Morris et al. (2015) adopted this paradigm to measure drowsiness during night driving. Longer PVT response times were observed as drivers became drowsier in the early hours of the morning. Consistent results were found in Kozak et al. (2005)’s study wherein the onset of drowsiness as measured by physiological and subjective ratings correlated with longer responses in the PVT.

The DRT was standardized in 2015 by the International Organization for Standardization to measure the increase in cognitive workload resulting from driver multitasking (ISO, 2015). It requires responding to an intermittent auditory, visual, or vibrotactile stimulus which is presented with an interstimulus interval that is drawn randomly from a uniform distribution of values between 3 and 5 seconds. Unlike the PVT which is typically administered as the sole task, the DRT is conceived to be administered concurrently with both a primary (i.e., driving) and a secondary task (e.g., interacting with a visual-manual, voice-based, or haptic interface) thus effectively serving as a tertiary performance task. Its duration is not set, and varies with that of the primary/secondary task at hand. Research has been conducted to quantify the potential cost of performing the DRT and, although it found a nonzero effect on cognitive workload (Biondi et al., 2021), its interference on the primary task performance is thought to be minimal especially during the completion of tasks in the real world (Castro et al., 2019; Stojmenova & Sodnik, 2018). Since its inception in 2015, the DRT has been widely adopted to measure the cognitive workload of using smartphone interfaces (Monk et al., 2023; Snider et al., 2021) and in-vehicle infotainment systems for navigation or media purposes (Lee et al., 2017; Strayer et al., 2015; Strayer Cooper, et al., 2017). This paradigm has also been used to quantify the residual cognitive cost of multitasking following the completion of the secondary task (Bowden et al., 2019; Strayer, Cooper, et al., 2017), and for measuring fluctuations in workload during driver-passenger conversations (Strayer et al., 2016).

## The Interpretation Conundrum

The success and popularity of stimulus detection tasks largely lie in their simple response time paradigm wherein one response (e.g., button press) is required to the presentation of one stimulus (e.g., a sound). With it, however, comes the issue of context-dependent data interpretation. To delineate this issue, for simplicity I will focus on response times alone, acknowledging however that my examination applies to task accuracy as well. I will also borrow from the theoretical approach of the inverted-U model of performance and arousal known as the Yerkes-Dodson law with its sole purpose of serving as a visual aid and while acknowledging its caveats (Hancock & Ganey, 2003).

**Figure 1.**
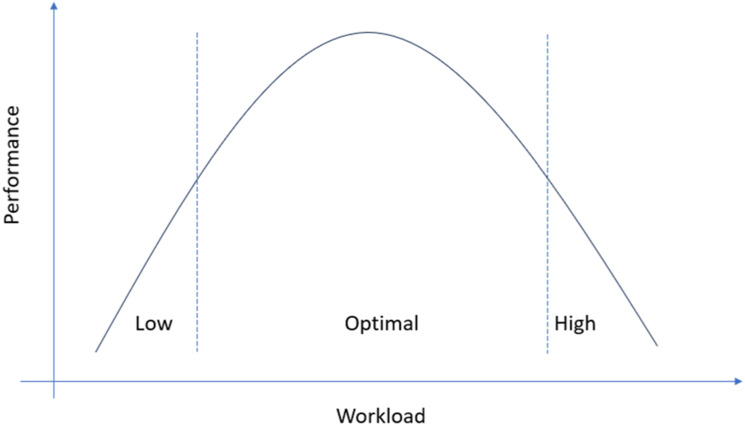
Inverted-U model of workload and performance.

The inverted-U model was originally conceived to better understand the relationship between physiological arousal and performance (Hebb, 1955) so that poor performance in a task follows either low or high arousal, and optimal performance occurs only at an intermediate arousal level. In Human Factors research, this model—and its right end-tail in particular—has been applied to describe the relationship between workload and performance in driving (Figure 1). For example, Curry et al. (2013), Reimer (2009), and Coughlin et al. (2011) used it to better understand the effect that driving under high levels of cognitive load has on decrements in driver behavior and road safety. More recently, the same conceptual framework—and its left end-tail in particular—has been borrowed in driving automation research to delineate the opposite scenario wherein low (not high) levels of workload result in poorer driving performance (Biondi et al., 2019, 2023; Rössger, 2015; Shupsky et al., 2020; Sibi et al., 2016). With the introduction of semi-automated systems that maintain control of the vehicle yet require the human driver to stay vigilant should a take-over be necessary (SAE level-2 and -3 systems; SAE, 2021), it is hypothesized that drivers will gradually lose engagement in the driving task (Strayer, Getty et al., 2017). In turn, this will slowly lead to a decrement in cognitive workload which, back to the inverted-U model, will lead to poorer performance during take-over scenarios.

The data interpretation conundrum begins when using a stimulus detection task as the primary source of information to ascribe declines in performance to either the left or right-end tail of the inverted-U curve. When inspected from the unique lens of this unidimensional paradigm, both low and high workload will in fact result in longer response times. Hamstrung, the researcher will then need to lean even more heavily on the theoretical construct of choice—underload or overload—to extricate themselves from the interpretation conundrum. This of course comes with clear unintended risks.

In a recent article, my coauthors and I used a detection task to measure vigilance decrements during the use of on-road semi-automated systems (Biondi et al., 2023). The task required participants to press a microswitch upon the presentation of a vibrotactile stimulus which occurred every 3–5 seconds. Following our decision to ground ourselves in the literature of user automation, we interpreted the longer response times observed during semi-automated driving not as indicative of greater workload (right-end tail of the inverted-U curve) but instead like the manifestation of lower workload and vigilance (left-end tail of the inverted-U curve). Zhang et al. (2021) found themselves in a similar bog when they, alongside the DRT, also used spectral EEG to measure changes in the driver’s state during monotonous automated driving. Greater alpha power—which is typically associated with a state of relaxation (Wang et al., 2013)—was found during automated driving. Together with longer responses in the DRT, data was interpreted as being the manifestation of driver drowsiness. Stapel et al. (2019) also used the DRT to measure changes in the driver’s state between manual and automated driving. Longer response times were found in automated driving mode, which they interpreted as being the result of greater workload. A consistent interpretation was given by (McDonnell et al., 2023) wherein, when analyzed within the context of the ISO standard, longer DRT response times during automated driving were accounted for as the manifestation of greater drivers’ workload. Theoretical wrangling aside, interpretation missteps have clear consequences on the scientific progress in Human Factors, especially when the exact same data can and will be accounted for in diametrically opposed fashions. For example, in the context of driving automation, the two hypotheses that operating automated systems will lead to a *reduction* in workload following greater driver disengagement or, instead, an *increase* in load resulting from the need to pay attention to both the system functioning and safety-related events have both equal merit when using a simple response task as the sole adjudicator. With this in mind, it becomes even more challenging to ascertain not only the true nature of the operator’s state but indeed the real implications—benefits or risks they may be—that automation use has on safety.

## Still in the Bog

The overview of the literature that I provided has hopefully persuaded the reader of the veracity of the interpretation conundrum when using a unidimensional tool like detection task paradigms for tackling multifaceted problems. As much as it requires a simple methodological setup, this approach may also demand taking sides when it comes to data interpretation, especially when it is taken as the primary source of information. With this comes two possible recommendations to Human Factors researchers and practitioners. The first is about taking into account the characteristics of the user and the task at hand. Given the known effect of practice and expertise on task performance (Hancock & Matthews, 2018; Parasuraman, 2014; Strayer et al., 2004), accounting for the user’s characteristics may provide additional information to disambiguate the etiology of the observed data patterns. Likewise, considering characteristics like task complexity and the single versus multi-tasking nature of the activity at hand may also facilitate the correct interpretation.

In keeping with the epistemic norms common of the scientific method, the second recommendation is about striving to gather additional evidence that both prove and refute the leading hypothesis rather than accepting it as summa veritas. Through strictly applying the approach known as data triangulation (Carayon et al., 2015), the Human Factors practitioner ought to gather data that unveils ulterior facets of task completion via the adoption of, for example, self-reported (e.g., POSWAT; Adrion, 1986) or physiological measures (e.g., EEG). This approach, which is already being adopted in some studies (Frazier et al., 2022; McDonnell, Imberger, et al., 2021; Scheutz et al., 2023; Zhang et al., 2021), should become more common practice in this discipline to help settle the debate once and for all. “Is the longer response times indicating of greater workload or drowsiness?” Let the debate continue, at least for now.

## Key Points

- This article tackles the conundrum of correct data interpretation when using stimulus detection tasks.
- The exact same detection task pattern may yield diametrically opposed data interpretations depending on the theoretical construct of choice.
- The evidence being discussed here highlights a clear limitation of detection tasks for determining the operator’s workload.
- Two recommendations are put forward to Human Factors researchers and practitioners.
